# Move-by-Move Dynamics of the Advantage in Chess Matches Reveals Population-Level Learning of the Game

**DOI:** 10.1371/journal.pone.0054165

**Published:** 2013-01-30

**Authors:** Haroldo V. Ribeiro, Renio S. Mendes, Ervin K. Lenzi, Marcelo del Castillo-Mussot, Luís A. N. Amaral

**Affiliations:** 1 Departamento de Física and National Institute of Science and Technology for Complex Systems, Universidade Estadual de Maringá, Maringá, Brazil; 2 Department of Chemical and Biological Engineering, Northwestern University, Evanston, Illinois, United States of America; 3 Departamento de Estado Sólido, Instituto de Física, Universidad Nacional Autónoma de México, Distrito Federal, México; 4 Northwestern Institute on Complex Systems (NICO), Northwestern University, Evanston, Illinois, United States of America; University of Maribor, Slovenia

## Abstract

The complexity of chess matches has attracted broad interest since its invention. This complexity and the availability of large number of recorded matches make chess an ideal model systems for the study of population-level learning of a complex system. We systematically investigate the move-by-move dynamics of the white player’s advantage from over seventy thousand high level chess matches spanning over 150 years. We find that the average advantage of the white player is positive and that it has been increasing over time. Currently, the average advantage of the white player is 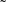
0.17 pawns but it is exponentially approaching a value of 0.23 pawns with a characteristic time scale of 67 years. We also study the diffusion of the move dependence of the white player’s advantage and find that it is non-Gaussian, has long-ranged anti-correlations and that after an initial period with no diffusion it becomes super-diffusive. We find that the duration of the non-diffusive period, corresponding to the opening stage of a match, is increasing in length and exponentially approaching a value of 15.6 moves with a characteristic time scale of 130 years. We interpret these two trends as a resulting from learning of the features of the game. Additionally, we find that the exponent 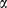 characterizing the super-diffusive regime is increasing toward a value of 1.9, close to the ballistic regime. We suggest that this trend is due to the increased broadening of the range of abilities of chess players participating in major tournaments.

## Introduction

The study of biological and social complex systems has been the focus of intense interest for at least three decades [1]. Elections [2], popularity [3], population growth [4], collective motion of birds [5] and bacteria [6] are just some examples of complex systems that physicists have tackled in these pages. An aspect rarely studied due to the lack of enough data over a long enough period is the manner in which agents learn the best strategies to deal with the complexity of the system. For example, as the number of scientific publication increases, researchers must learn how to choose which papers to read in depth [7]; while in earlier times word-of-mouth or listening to a colleague’s talk were reliable strategies, nowadays the journal in which the study was published or the number of citations have become, in spite of their many caveats, indicators that seem to be gaining in popularity.

In order to understand how population-level learning occurs in the “real-word,” we study it here in a model system. Chess is a board game that has fascinated humans ever since its invention in sixth-century India [8]. Chess is an extraordinary complex game with 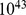 legal positions and 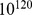 distinct matches, as roughly estimated by Shannon [9]. Recently, Blasius and Tönjes [10] have showed that scale-free distributions naturally emerge in the branching process in the game tree of the first game moves in chess. Remarkably, this breadth of possibilities emerges from a small set of well-defined rules. This marriage of simple rules and complex outcomes has made chess an excellent test bed for studying cognitive processes such as learning [11], [12] and also for testing artificial intelligence algorithms such as evolutionary algorithms [13].

The very best chess players can foresee the development of a match 10–15 moves into the future, thus making appropriate decisions based on his/her expectations of what his opponent will do. Even though super computers can execute many more calculations and hold much more information in a quickly accessible mode, it was not until heuristic rules were developed to prune the set of possibilities that computers became able to consistently beat human players. Nowadays, even mobile chess programs such as Pocket Fritz™ (http://chessbase-shop.com/en/products/pocket_fritz_4) have a Elo rating [14] of 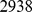 which is higher than the current best chess player (Magnus Carlsen with a Elo rating of 2835 – http://fide.com).

The ability of many chess engines to accurately evaluate the strength of a position enables us to numerically evaluate the move-by-move white player advantage 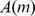 and to determine the evolution of the advantage during the course of a chess match. In this way, we can probe the patterns of the game to a degree not before possible and can attempt to uncover population-level learning in the historical evolution of chess match dynamics. Here, we focus on the dynamical aspects of the game by studying the move-by-move dynamics of the white player’s advantage 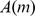 from over seventy thousand high level chess matches.

**Figure 1:**
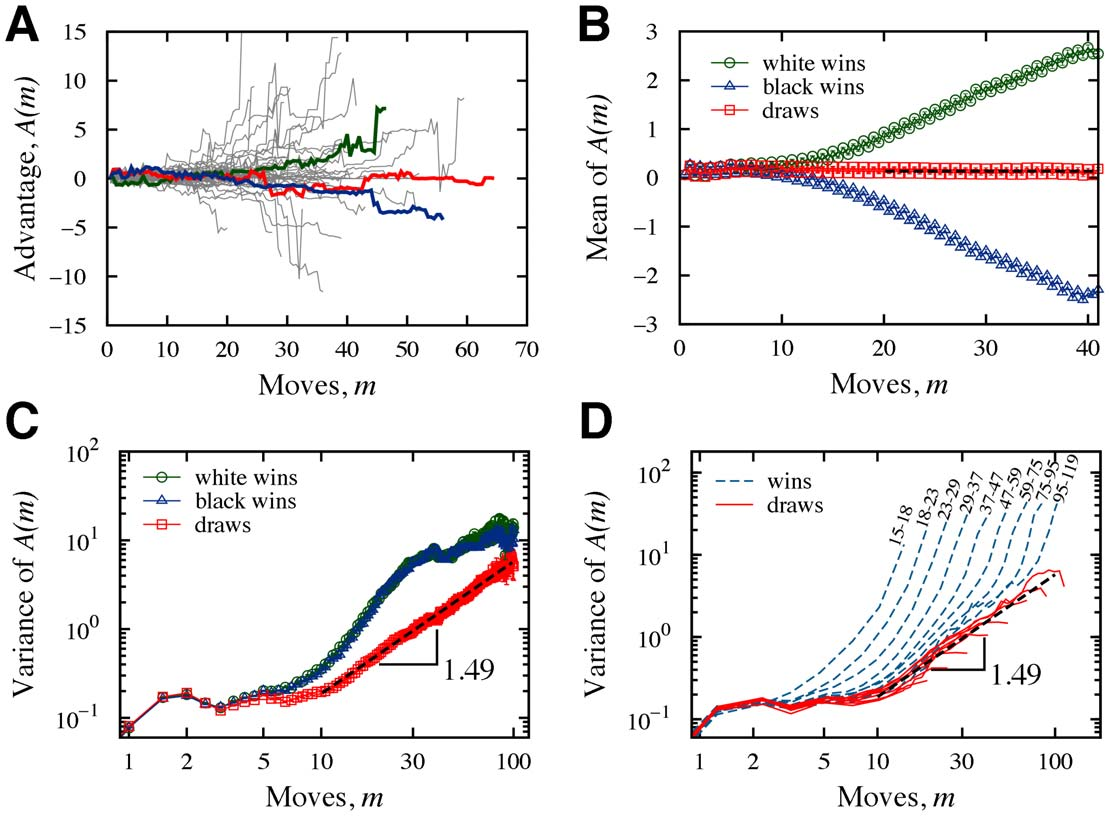
Diffusive dynamics of white player’s advantage. (A) Evolution of the advantage 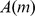 for 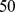 matches selected at random. We highlight the trajectories from three World Chess Championship matches: the 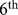 match between Anand (playing white) and Kramnik in 2008 (green line), the 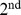 match between Karpov (playing white) and Kasparov in 1985 (red line), and the 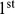 match between Spassky (playing white) and Petrosian in 1969 (blue line). (B) Mean value of the advantage as a function of move number for matches ending in draws (squares), white wins (circles) and black wins (triangles). Note the systematically alternating values and the initial positive values of these means for all outcomes. For white wins, the mean advantage increases with 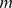, while for black wins it decreases. For draws, the mean advantage is approximately a positive constant. We estimated the advantage of playing white to be 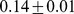 and horizontal dashed line represents this value. (C) Variance of the advantage as a function of move number for matches ending in draws (squares) and white wins (circles) and black wins (triangles). Note the very similar profile of the variance for white and black wins. Note also that there is practically no diffusion for the initial 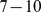 moves, corresponding to the opening period, a very well studied stage of the game. After the opening stage, the trajectories exhibit a faster than diffusive spreading. For draws, we find this second regime (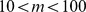) to be superdiffusive and characterized by an exponent 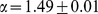, as shown by the dashed line. For wins, the variance presents a more complex behavior. For 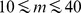 the variance increases faster than ballistic (hyper-diffusion), but for later stages it displays a behavior similar to that found for draws. (D) Variance of advantage evaluated after grouping the matches by length and outcome. For draws (continuous lines), the different match lengths do not change the power-law dependence of the variance. For wins (dashed lines), the variance systematically approaches the profile obtained for draws as the matches becomes longer. We further note the existence of a very fast diffusive regime for the latest moves of each grouping.

We have accessed the portable game notation (PGN) files of 73,444 high level chess matches made free available by PGN Mentor™ (http://www.pgnmentor.com). These data span the last two centuries of the chess history and cover the most important worldwide chess tournaments, including the World Championships, Candidate Tournaments, and the Linares Tournaments (see Table S1). White won 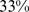 of these matches, black won 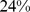 and 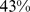 ended up with in a draw. For each of these 73,444 matches, we estimated 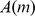 using the Crafty™ [15] chess engine which has an Elo rating of 2950 (see Methods Section A). The white player advantage 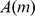 takes into account the differences in the number and the value of pieces, as well as the advantage related to the placement of pieces. It is usually measured in units of pawns, meaning that in the absence of other factors, it varies by one unit when a pawn (the pieces with lowest value) is captured. A positive value indicates that the white player has the advantage and a negative one indicates that the black player has the advantage. Figure 1A illustrates the move dependence of 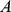 for 50 matches selected at random from the data base. Intriguingly, 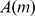 visually resembles the “erratic” movement of diffusive particles.

## Results

We first determined how the mean value of the advantage depends on the move number 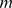 across all matches with the same outcome (Fig. 1B). We observed an oscillatory behavior around a positive value with a period of 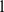 move for both match outcomes. This oscillatory behavior reflects the natural progression of a match, that is, the fact that the players alternate moves. Not surprisingly, for matches ending in a draw the average oscillates around an almost stable value, while for white wins it increases systematically and for black wins it decreases systematically.

Figure 1B suggests an answer to an historical debate among chess players: Does playing white yield an advantage? Some players and theorists argue that because the white player starts the game, white has the “initiative, ” and that black must endeavor to equalize the situation. Others argue that playing black is advantageous because white has to reveal the first move. Chess experts usually mention that white wins more matches as evidence of this advantage. However, the winning percentage does not indicate the magnitude of this advantage. In our analysis, we not only confirm the existence of an advantage in playing white, but also estimate its value as 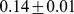 by averaging the values of the mean for matches ending in draws.

We next investigated the diffusive behavior by evaluating the dependence of the variance of 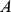 on the move number 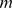 (Fig. 1C). After grouping the matches by match outcome, we observed for all outcomes that there is practically no diffusion during the initial moves. These moves correspond to the opening period of the match, a stage very well studied and for which there are recognized sequences of moves that result in balanced positions. After this initial stage, the variance exhibits an anomalous diffusive spreading. For matches ending in a draw, we found a super-diffusive regime (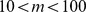) that is described by a power law with an exponent 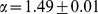. We note the very similar profile of the variance of matches ending in white or black wins.

Matches ending in a win display an hyper-diffusive regime (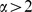)– a signature of nonlinearity and out-of-equilibrium systems [16]. In fact, the behavior for matches ending in wins is quite complex and dependent on the match length (Fig. 1D). While grouping the matches by length does not change the variance profile of draws, for wins it reveals a very interesting pattern: As the match length increases the variance profile become similar to the profile of draws, with the only differences occurring in the last moves. This result thus suggests that the behavior of the advantage of matches ending in a win is very similar to a draw. The main difference occurs in last few moves where an avalanche-like effect makes the advantage undergo large fluctuations.

### Historical Trends

**Figure 2:**
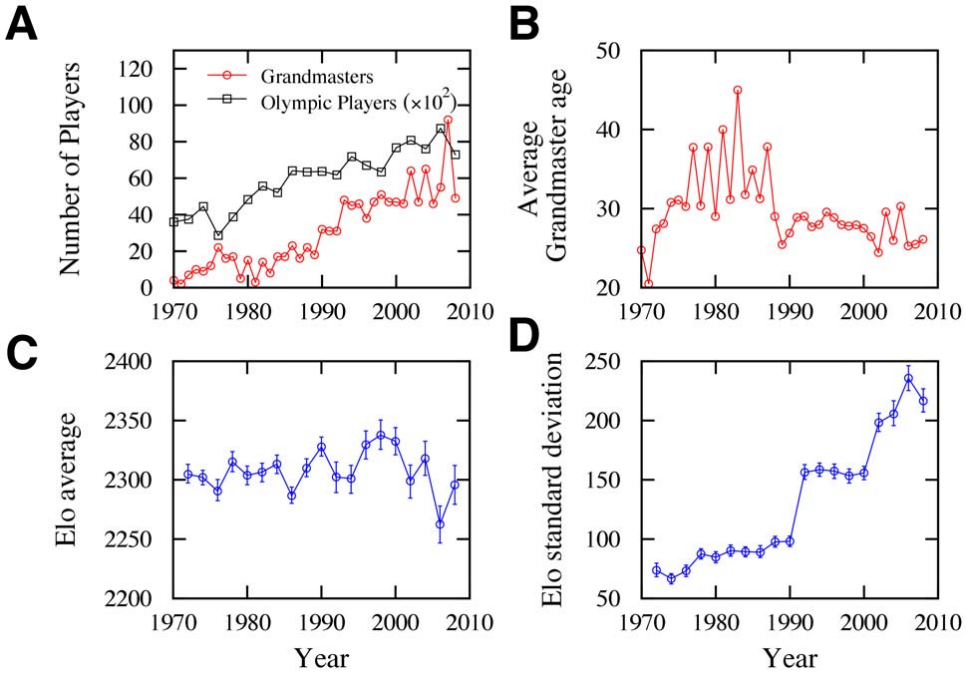
Historical changes in chess player demographics. (A) Number of new Chess Grandmaster awarded annually by the world chess organization (http://fide.com) and the number of players who have participated in the Chess Olympiad (http://www.olimpbase.org) since 1970. Note the increasing trends in these quantities. (B) Average players’ age when receiving the Grandmaster title. (C) Average Elo rating and (D) standard deviation of the of Elo rating of players who have participated in the Chess Olympiad. Note the nearly constant value of the average, while the standard deviation has increased dramatically.

Chess rules have been stable since the 19th century. This stability increased the game popularity (Fig. 2A) and enabled players to work toward improving their skill. A consequence of these efforts is the increasing number of Grandmasters – the highest title that a player can attain – and the decreasing average player’s age for receiving this honor (Figs. 2A and 2B). Intriguingly, the average player’s fitness (measured as the Elo rating [14]) in Olympic tournaments has remained almost constant, while the standard deviation of the player’s fitness has increased fivefold (Figs. 2C and 2D). These historical trends prompt the question of whether there has been a change in the diffusive behavior of the match dynamics over the last 150 years.

**Figure 3:**
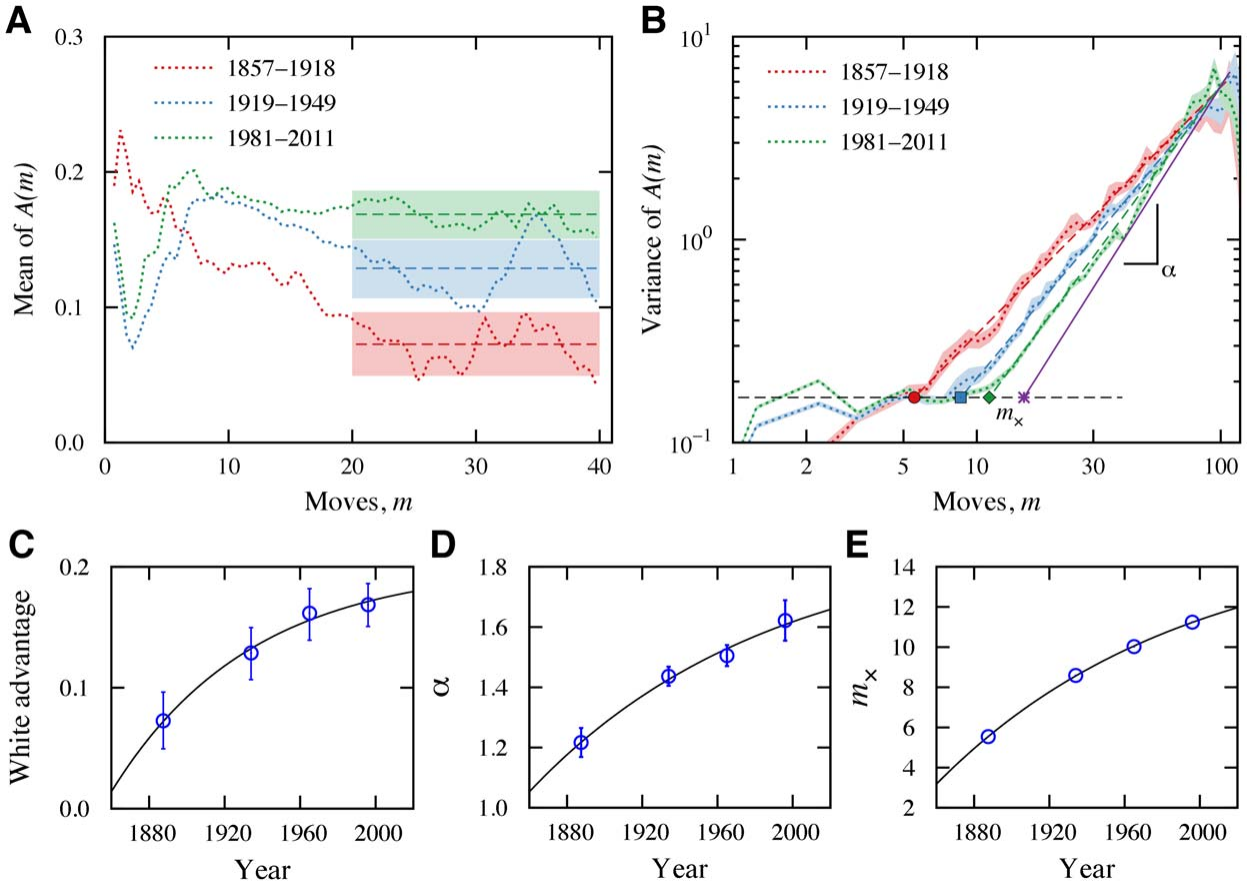
Historical trends in the dynamics of highest level chess matches. (A) Mean value of the advantage of matches ending a draw for three time periods. These curves were smoothed by using moving averaging over windows of size 2. The horizontal lines are the averaged values of the mean for 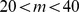 and the shaded regions are 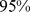 confidence intervals for these averaged values. (B) Variance of the advantage of matches ending a draw for three time periods. The shaded regions are 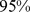 confidence intervals for the variance and the colored dashed lines indicate power law fits to each data set. The horizontal dashed line represents the average variance for the most recent data set and for 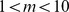. Note the systematic increase of 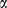 and of the number of moves in the opening. The symbols on this line indicate the values of 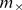, the number of moves at which the diffusion of the advantage changes behavior. The rightmost symbol represent the extrapolated maximum value 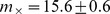. (C) Time evolution of the white player advantage for matches ending in draws. The solid line represents an exponential approach to an asymptotic value. The estimated plateau value is 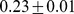 pawns and the characteristic time is 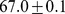 years. Time evolution of (D) the exponent 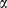 and (E) the crossover move 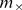. The solid lines are fits to exponential approaches to the asymptotic values 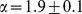 and 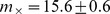. The estimated characteristic times for convergence are 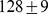 years for the diffusive exponent and 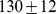 years for the crossover move. Based on the conjecture that 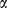 and 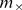 are approaching limiting values, we plotted a continuous line in Fig 3B to represent this limiting regime.

To answer this question, we investigated the evolution of the profile of the mean advantage for different periods (Fig. 3A). For easier visualization, we applied a moving averaging with window size two to the mean values of 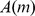. The horizontal lines show the average values of the means for 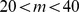 and the shaded areas are 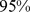 confidence intervals obtained via bootstrapping. The average values are significantly different, showing that the baseline white player advantage has increased over the last 150 years. We found that this increase is well described by an exponential approach with a characteristic time of 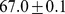 years to an asymptotic value of 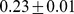 pawns (Fig. 3C). Our results thus suggest that chess players are learning how to maximize the advantage of playing white and that this advantage is bounded.

Next, we considered the time evolution of the variance for matches ending in draws (Fig. 3B). Surprisingly, 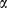 seems to be approaching a value close to that for a ballistic regime. We found that the exponent 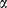 follows an exponential approach with a characteristic time of 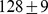 years to the asymptote 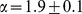 (Fig. 3D). We surmise that this trend is directly connected to an increase in the typical difference in fitness among players. Specifically, the presence of fitness in a diffusive process has been shown to give rise to ballistic diffusion [17]. For an illustration of how differences in fitness are related to a ballistic regime (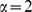), assume that
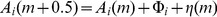
(1)describes the advantage of the white player in a match 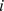, where the difference in fitness between two players is 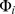 and 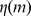 is a Gaussian variable. 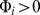 yields a positive drift in 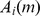 thus modeling a match where the white player is better. Assuming that the fitness 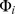 is drawn from a distribution with finite variance 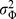, it follows that



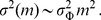
(2)Thus, 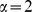. In the case of chess, the diffusive scenario is not determined purely by the fitness of players. However, differences in fitness are certainly an essential ingredient and thus Eq.(1) can provide insight into the data of Fig. 3D by suggesting that the typical difference in skill between players has been increasing.

A striking feature of the results of Fig. 3B is the drift of the crossover move 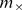 at which the power-law regime begins. We observe that 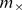 is exponentially approaching an asymptote at 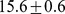 moves with a characteristic time of 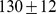 years (Fig. 3E). Based on the existence of limiting values for 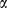 and 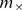, we plot in Figure 3B an extrapolated power law to represent the limiting diffusive regime (continuous line). We have also found that the distributions of the match lengths for wins and draws display exponential decays with characteristics lengths of 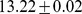 moves for draws and 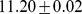 moves for wins. Moreover, we find that these characteristic lengths have changed over the history of chess. For matches ending in draws, we observed a statistically significant growth of approximately 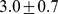 moves per century. For wins, we find no statistical evidence of growth and the characteristic length can be approximated by a constant mean of 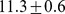 moves (Fig. S1).

A question posed by the time evolution of these quantities is whether the observed changes are due to learning by chess players over time or due to a secondary factor such as changes in the organization of chess tournaments. In order to determine the answer to this question, we analyze the type of tournaments included in the database. We find that 88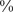 of the tournaments in the database use “round-robin” pairing (all-play-all) and that there has been an increasing tendency to employ this pairing scheme (Fig. S2). In order to further strengthen our conclusions, we analyze the matches in the database obtained by excluding tournaments that do not use round-robin pairing. This procedure has the advantage that it reduces the effect of non-randomness sampling. As shown in Fig. S3, this procedure does not change the results of our analyses.

**Figure 4:**
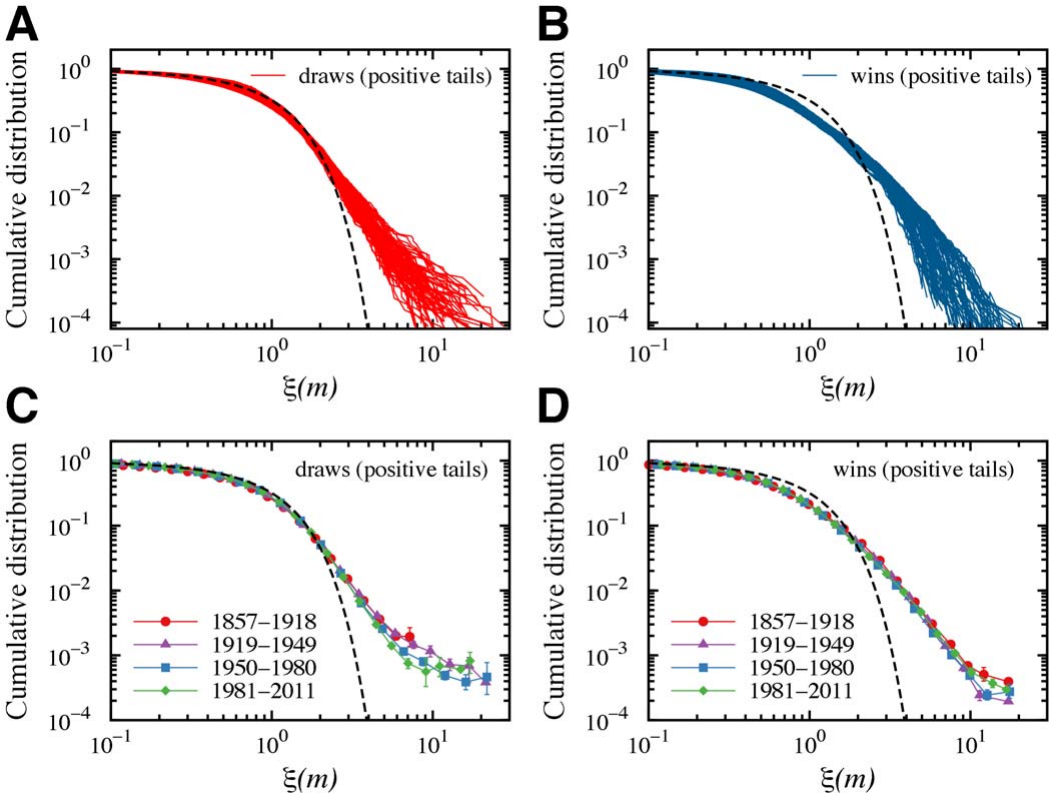
Scale invariance and non-Gaussian properties of the white player’s advantage. Positive tails of the cumulative distribution function for the normalized advantage 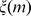 for matches ending in (A) draws and (B) wins. Each line in these plots represents a distribution for a different value of 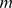 in the range 10 to 70. By match outcome, the distributions for different values of 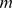 exhibit a good data collapse with tails that decay slower than a Gaussian distribution (dashed line). Average cumulative distribution for matches ending in (C) draws and (D) wins for four time periods. We estimated the error bars using bootstrapping. These data support the hypothesis of scaling, that is, the distributions follow a universal non-Gaussian functional form. The negative tails present a very similar shape (see Fig. S4).

We next studied the distribution profile of the advantage. We use the normalized advantage
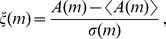
(3)where 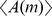 is the mean value of advantage after 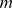 moves and 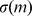 is the standard-deviation. Figures 4A and 4B show the positive tails of the cumulative distribution of 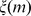 for draws and wins for 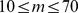. We observe the good data collapse, which indicates that the advantages are statistically self-similar, since after scaling they follow the same universal distribution. Moreover, Figs. 4D and 4E show that the distribution profile of the normalized advantage is quite stable over the last 150 years. These distributions obey a functional form that is significantly different from a Gaussian distribution (dashed line in the previous plots). In particular, we observe a more slowly decaying tail, showing the existence of large fluctuations even for matches ending in draws.

**Figure 5:**
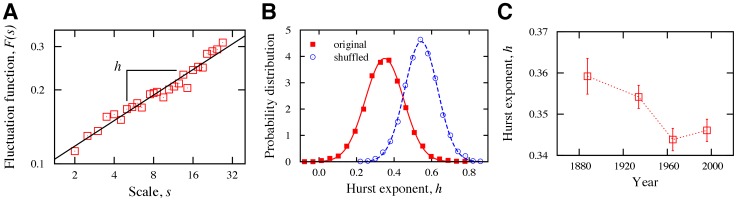
Long-range correlations in white player’s advantage. (A) Detrended fluctuation analysis (DFA, see Methods Section B) of white player’s advantage increments, that is, 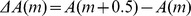, for a match ended in a draw and selected at random from the database. For series with long-range correlations, the relationship between the fluctuation function 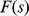 and the scale 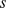 is a power-law where the exponent is the Hurst exponent 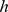. Thus, in this log-log plot the relationship is approximated by a straight line with slope equal to 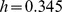. In general, we find all these relationships to be well approximated by straight lines with an average Pearson correlation coefficient of 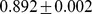. (B) Distribution of the estimated Hurst exponent 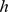 obtained using DFA for matches longer than 50 moves that ended in a draw (squares). The continuous line is a Gaussian fit to the distribution with mean 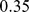 and standard-deviation 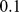. Since 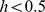, it implies an anti-persistent behavior (see Fig. 1B). We have also evaluated the distribution of 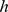 using the shuffled version of these series (circles). For this case, the dashed line is a Gaussian fit to the data with mean 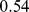 and standard-deviation 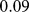. Note that the shuffled procedure removed the correlations, confirming the existence of long-range correlations in 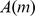. (C) Historical changes in the mean Hurst exponent 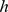. Note the significantly small values of 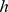 in recent periods, showing that the anti-persistent behavior has increased for more recent matches.

Another intriguing question is whether there is memory in the evolution of the white player’s advantage. To investigate this hypothesis, we consider the time series of advantage increments 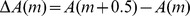 for all 5,154 matches ending in a draw that are longer than 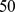 moves. We used detrended fluctuation analysis (DFA, see Methods Section B) to obtain the Hurst exponent for each match (Fig. 5A). We find 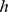 distributed around 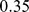 (Fig. 5B) which indicates the presence of long-range anti-correlations in the evolution of 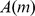. A value of 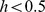 indicates the presence of an anti-persistent behavior, that is, the alternation between large and small values of 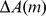 occurs much more frequently than by chance. This result also agrees with the oscillating behavior of the mean advantage (Fig. 1B). We also find that the Hurst exponent 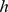 has evolved over time (Fig. 5C). In particular, we note that the anti-persistent behavior has statistically increased for the recent two periods, indicating that the alternating behavior has intensified in this period. We have found a very similar behavior for matches ending in wins after removing the last few moves in the match (Fig. S5).

## Discussion

We have characterized the advantage dynamics of chess matches as a self-similar, super-diffusive and long-ranged-memory process. Our investigation provides insights into the complex process of creating and disseminating knowledge of a complex system at the population-level. By studying 150 years of high level chess, we presented evidence that the dynamics of a chess have evolved over time in such a way that it appears to be approaching a steady-state. The baseline advantage of the white player, the cross-over move 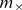, and the diffusive exponent 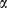 are exponentially approaching asymptotes with different characteristic times. We hypothesized that the evolution of 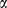 are closely related to an increase in the difference of fitness among players, while the evolution of the baseline advantage of white player indicates that players are learning better ways to explore this advantage. The increase in the cross-over move 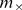 suggest that the opening stage of a match is becoming longer which may also be related to a collective learning process. As discussed earlier, hypothesized historical changes in pairing scheme during tournaments cannot explain these findings.

## Methods

### Estimating 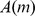


The core of a chess program is called the chess engine. The chess engine is responsible for finding the best moves given a particular arrangement of pieces on the board. In order to find the best moves, the chess engine enumerates and evaluates a huge number of possible sequences of moves. The evaluation of these possible moves is made by optimizing a function that usually defines the white player’s advantage. The way that the function is defined varies from engine to engine, but some key aspects, such as the difference of pondered number of pieces, are always present. Other theoretical aspects of chess such as mobility, king safety, and center control are also typically considered in a heuristic manner. A simple example is the definition used for the GNU Chess program in 1987 (see http://alumni.imsa.edu/ 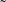
stendahl/comp/txt/gnuchess.txt). There are tournaments between these programs aiming to compare the strength of different engines. The results we present were all obtained using the Crafty™ engine [15]. This is a free program that is ranked 24th in the Computer Chess Rating Lists (CCRL - http://www.computerchess.org.uk/ccrl). We have also compared the results of subsets of our database with other engines, and the estimate of the white player advantage proved robust against those changes.

### DFA

DFA consists of four steps [18], [19]:

- We define the profile

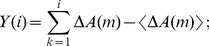

- We cut 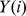 into 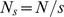 non-overlapping segments of size 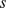, where 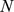 is the length of the series;
- For each segment a local polynomial trend (here, we have used linear function) is calculated and subtracted from 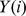, defining 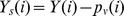, where 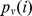 represents the local trend in the 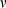-th segment;
- We evaluate the fluctuation function

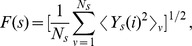
where 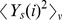 is mean square value of 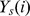 over the data in the 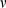-th segment.

If 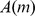 is self-similar, the fluctuation function 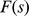 displays a power-law dependence on the time scale 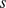, that is, 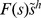, where 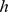 is the Hurst exponent.

## Supporting Information

Figure S1
**Historical trends in match lengths.** Cumulative distribution function for the match lengths ending in **(A)** draws and wins **(B)**. Both distributions display an exponential decay with characteristic lengths 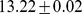 for draws and 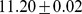 for wins. **(C)** Cumulative distribution function for the match lengths ending white wins (circles) and black wins (triangles). Note that both distributions are almost indistinguishable. **(D)** Changes in the characteristic game length 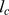 over time. For draws (squares), we observe a statistically significant growth of approximately 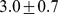 moves per century (red line). For wins (circles), we find that 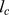 is approximately constant with mean value 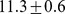 (green line).(TIF)

Figure S2 Percentage of tournaments that employ the round-robin (all-play-all) pairing scheme. Note the increase in the fraction of tournaments employing round-robin pairing scheme.
(TIF)

Figure S3 **The effect of excluding tournaments using the swiss-pairing scheme on the historical trends reported in** **Fig. 3** **.** It is visually apparent that excluding data from those tournaments does not significantly change our results. Thus, temporal changes in the pairing schemes used in chess tournaments can not explain our findings.
(TIF)

Figure S4
**Scale invariance and non-Gaussian properties of the white player’s advantage.** Negative tails of the cumulative distribution function for the normalized advantage 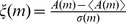 for matches ending in (A) draws and (B) wins. Each line in these plots represents a distribution for a different value of 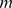 in the range 10 to 70. For match outcome, the distributions for different values of 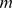 exhibit a good data collapse with tails that decay slower than a Gaussian distribution (dashed line). Average cumulative distribution for matches ending in (C) draws and (D) wins for four time periods. We estimated the error bars using bootstrapping.(TIF)

Figure S5
**Match outcome and long-range correlations in the white player’s advantage.** Distribution of the estimated Hurst exponent 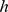 obtained using DFA for matches longer than 50 moves that ended in draws (squares), wins (circles) and wins after dropping the five last moves of each match. The continuous line is a Gaussian fit to the distribution for draws with mean 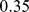 and standard-deviation 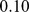. For wins, the mean value of 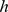 is 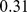 and the standard-deviation is 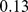. Note that after dropping the five last moves the distribution of 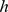 for wins becomes very close to distribution obtained for draws. The mean value in this last case is 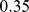 and the standard-deviation is 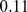.(TIF)

Table S1 **Full description of our chess database.** This table show all the tournaments that comprise our data base. The PGN files are free available at http://www.pgnmentor.com/files.html. Specifically, the files we have used are those grouped under sections “Tournaments”, “Candidates and Interzonals” and “World Championships”.
(PDF)
