# Supplementary figures and images for: Move-by-Move Dynamics of the Advantage in Chess Matches Reveals Population-Level Learning of the Game
Source: PLoS One. 2013 Jan 30;8(1):e54165. doi: 10.1371/journal.pone.0054165 (PMC3559554; doi:10.1371/journal.pone.0054165)

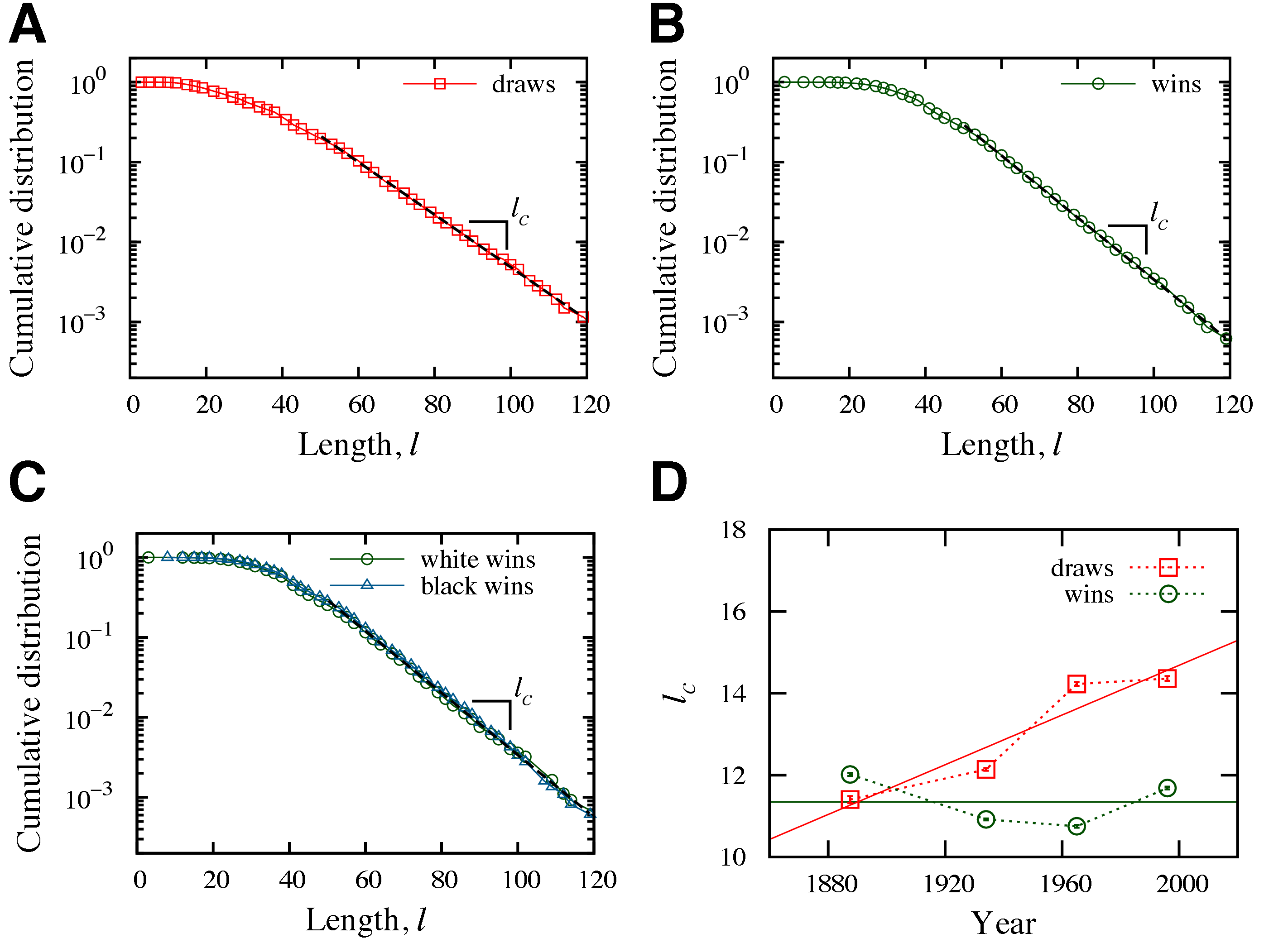

Supplement: Figure S1 — Historical trends in match lengths. Cumulative distribution function for the match lengths ending in (A) draws and wins (B). Both distributions display an exponential decay with characteristic lengths for draws and for wins. (C) Cumulative distribution function for the match lengths ending white wins (circles) and black wins (triangles). Note that both distributions are almost indistinguishable. (D) Changes in the characteristic game length over time. For draws (squares), we observe a statistically significant growth of approximately moves per century (red line). For wins (circles), we find that is approximately constant with mean value (green line). (TIF) [file pone.0054165.s001.tif]

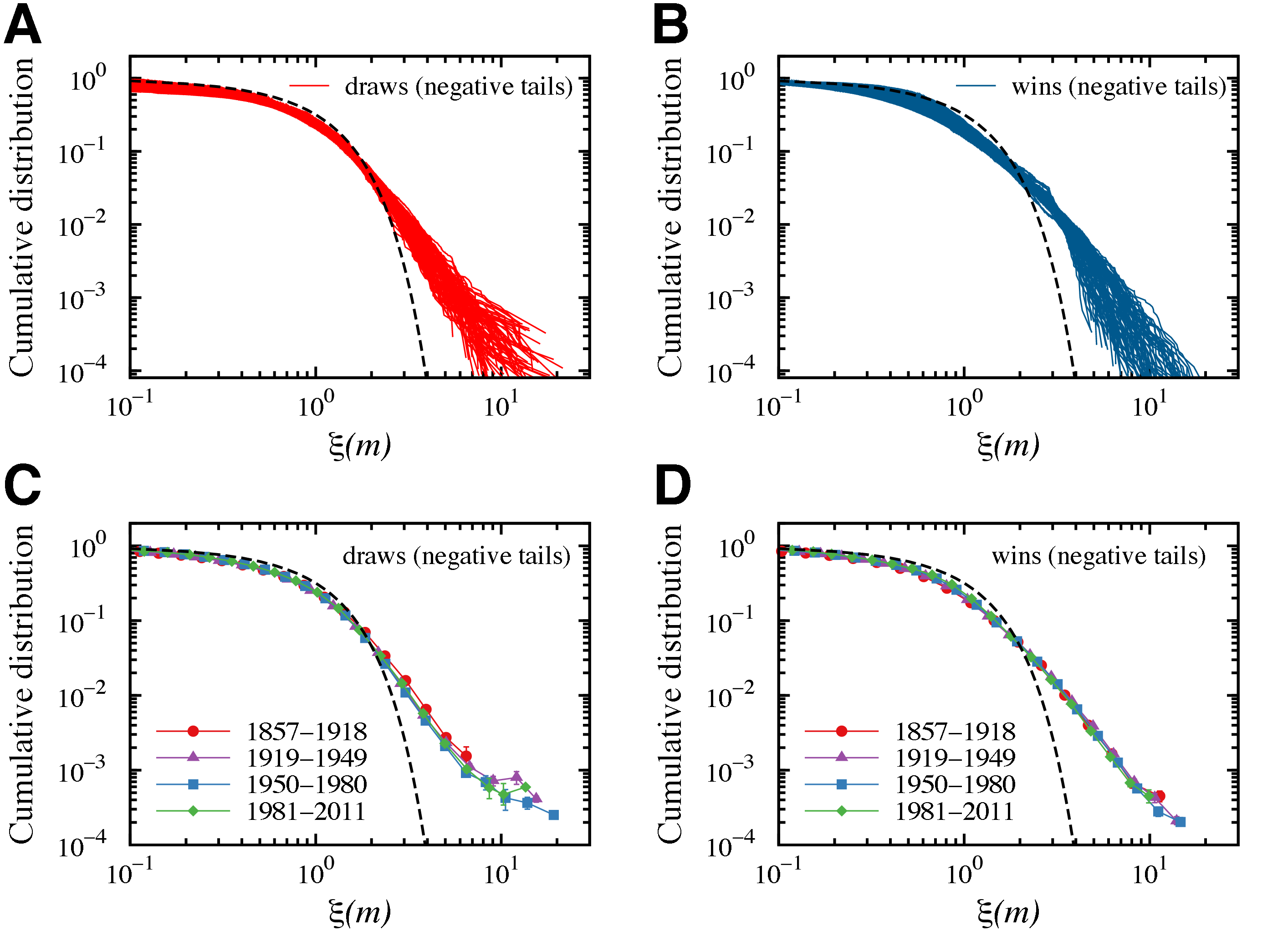

Supplement: Figure S2 — Percentage of tournaments that employ the round-robin (all-play-all) pairing scheme. Note the increase in the fraction of tournaments employing round-robin pairing scheme. (TIF) [file pone.0054165.s002.tif]

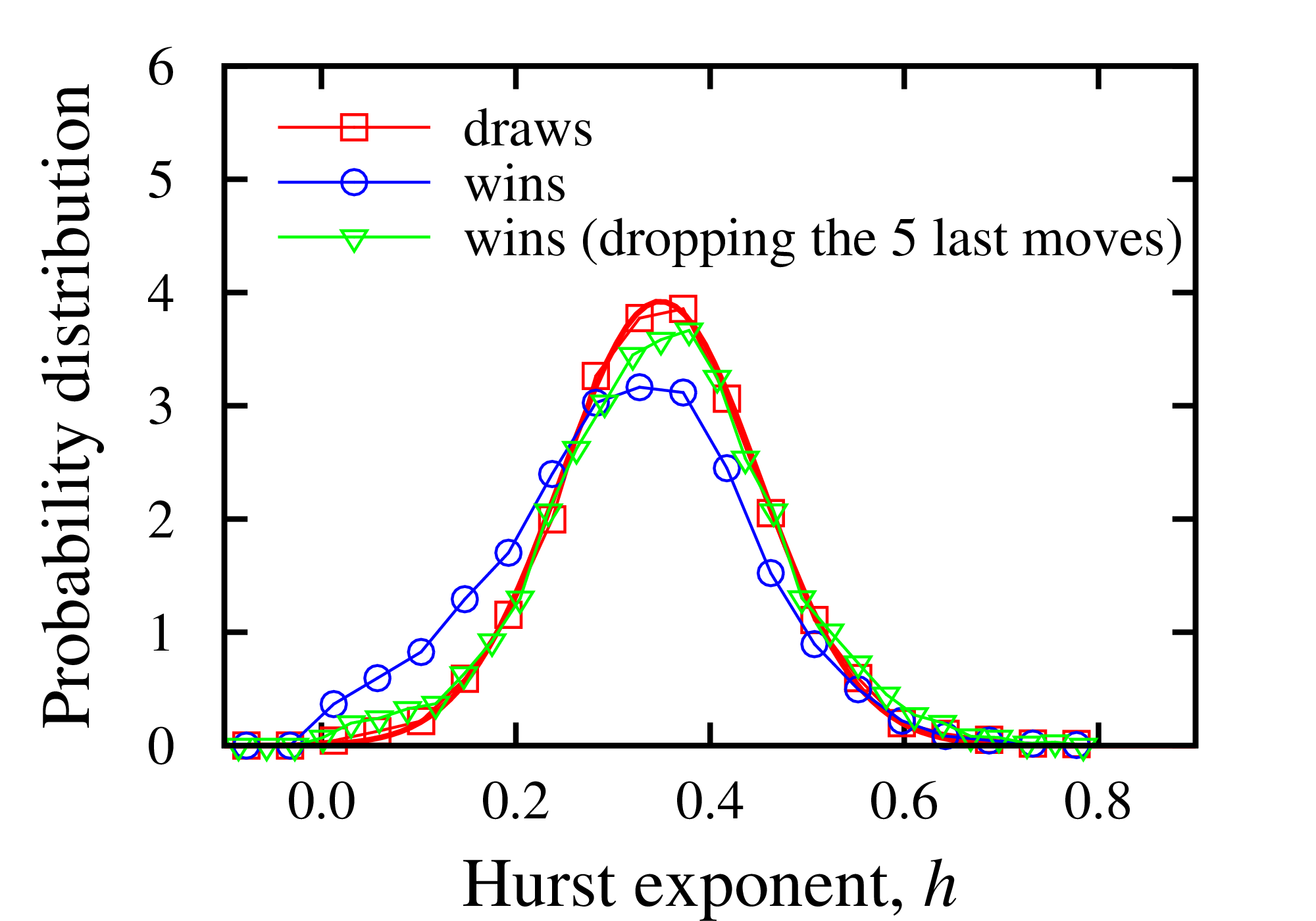

Supplement: Figure S3 — The effect of excluding tournaments using the swiss-pairing scheme on the historical trends reported in Fig. 3 . It is visually apparent that excluding data from those tournaments does not significantly change our results. Thus, temporal changes in the pairing schemes used in chess tournaments can not explain our findings. (TIF) [file pone.0054165.s003.tif]

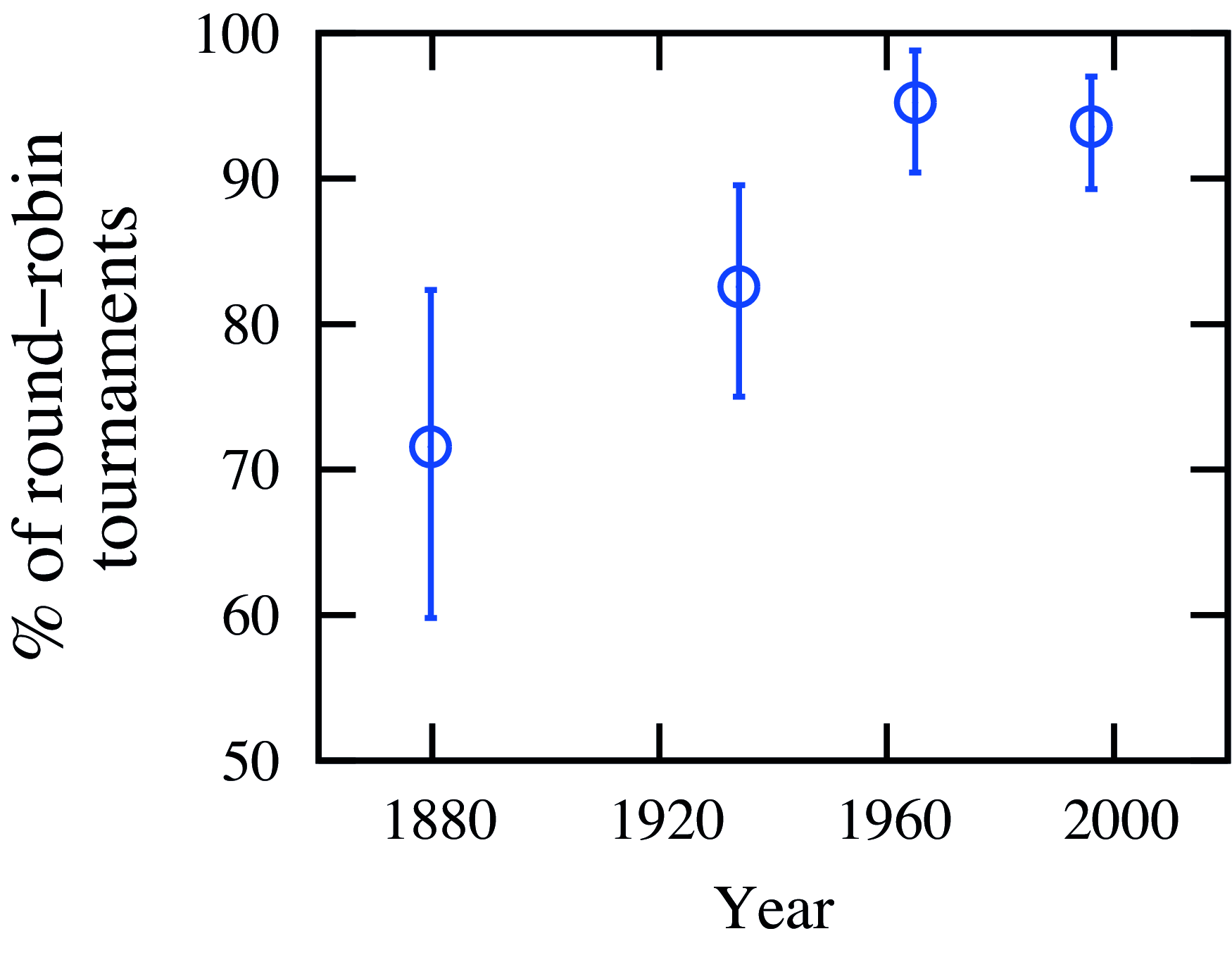

Supplement: Figure S4 — Scale invariance and non-Gaussian properties of the white player’s advantage. Negative tails of the cumulative distribution function for the normalized advantage for matches ending in (A) draws and (B) wins. Each line in these plots represents a distribution for a different value of in the range 10 to 70. For match outcome, the distributions for different values of exhibit a good data collapse with tails that decay slower than a Gaussian distribution (dashed line). Average cumulative distribution for matches ending in (C) draws and (D) wins for four time periods. We estimated the error bars using bootstrapping. (TIF) [file pone.0054165.s004.tif]

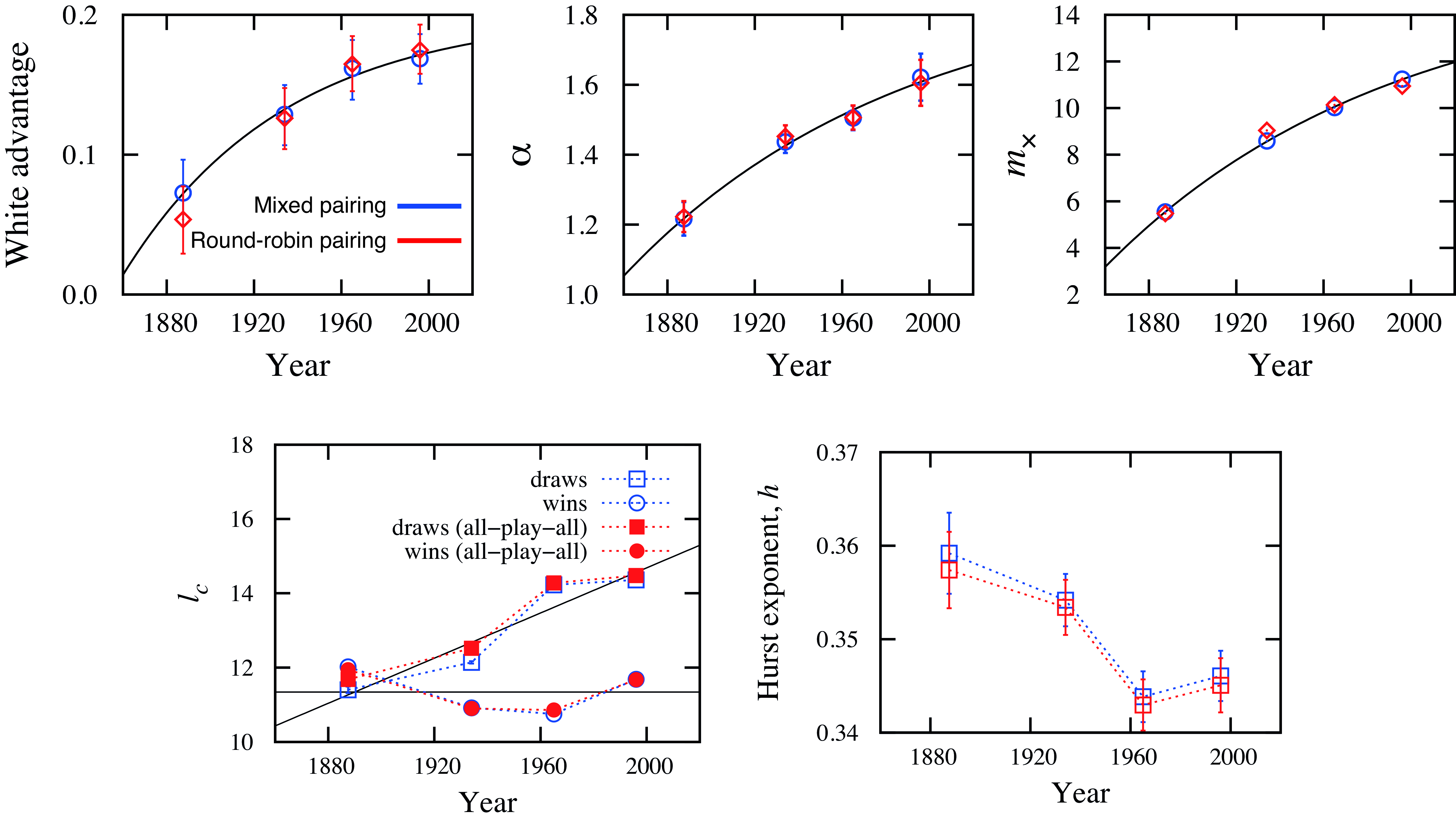

Supplement: Figure S5 — Match outcome and long-range correlations in the white player’s advantage. Distribution of the estimated Hurst exponent obtained using DFA for matches longer than 50 moves that ended in draws (squares), wins (circles) and wins after dropping the five last moves of each match. The continuous line is a Gaussian fit to the distribution for draws with mean and standard-deviation . For wins, the mean value of is and the standard-deviation is . Note that after dropping the five last moves the distribution of for wins becomes very close to distribution obtained for draws. The mean value in this last case is and the standard-deviation is . (TIF) [file pone.0054165.s005.tif]
