# Supplementary material for: Move-by-Move Dynamics of the Advantage in Chess Matches Reveals Population-Level Learning of the Game
Source: PLoS One. 2013 Jan 30;8(1):e54165. doi: 10.1371/journal.pone.0054165 (PMC3559554; doi:10.1371/journal.pone.0054165)
Supplement: Table S1 — Full description of our chess database. This table show all the tournaments that comprise our data base. The PGN files are free available at http://www.pgnmentor.com/files.html. Specifically, the files we have used are those grouped under sections “Tournaments”, “Candidates and Interzonals” and “World Championships”. (PDF) [file pone.0054165.s006.pdf]

Table S1. Full description of our chess database. This table show all the tournaments that comprise our data base. The PGN files are free available at `http://www.pgnmentor.com/files.html`. Specifically, the files we have used are those grouped under sections "Tournaments", "Candidates and Interzonals" and "World Championships".

**Tournament**	**Years**
World Championships	
FIDE Championship	1996,1998-2000,2002,2004-2008,2010
PCA Championship	1993,1995
World Championship	1886,1889,1890,1892,1894,1896,1907-1910,1921,1927,1929,1934,1935, 1937,1948,1951,1954,1957,1958,1960,1961,1963,1966,1969,1972,1978, 1981,1985,1987,1990,1993
Candidates and Interzonals	
Candidates	1950,1953,1959,1962,1965,1968,1971,1974,1980,1983,1985,1990,1994
Interzonals	1948,1952,1955,1958,1962,1964,1967,1970,1973,1976,1979,1982,1985, 1987,1990,1993
WCC Qualifier	1998,2002,2007,2009
PCA Candidates	1994
PCA Qualifier	1993
World Cup	2005
Open Tournaments	
AVRO	1938
Aachen	1868
Altona	1869,1872
Amsterdam	1889,1920,1936,1976-1981,1985,1987,1988,1991,1993-1996
Bad	1977
BadElster	1937-1939
BadHarzburg	1938,1939
BadKissingen	1928,1980,1981
BadNauheim	1935-1937
BadNiendorf	1927
BadOeynhausen	1922
BadPistyan	1912,1922
Baden	1870,1925,1980
Barcelona	1929,1935,1989
Barmen	1869,1905
Belfort	1988
Belgrade	1964,1993,1997
Berlin	1881,1897,1907,1920,1926
Bermuda	2005
Bern	1932
Beverwijk	1967
Biel	1992,1997,2004,2006,2007
Bilbao	2009
Birmingham	1858
Bled	1931,1961,1979
Bournemouth	1939
Bradford	1888,1889

Continued on next page

Table S1 – continued from previous page

Open	Years
Breslau	1889,1912,1925
Bristol	1861
Brussels	1986-1988
Bucharest	1953
Budapest	1896,1913,1921,1926,1929,1940,1952,2003
Budva	1967
Buenos Aires	1939,1944,1960,1970,1980,1994
Bugojno	1978,1980,1982,1984,1986
Cambridge	1904
Cannes	2002
Carlsbad	1907
Carrasco	1921,1938
Chicago	1874,1982
Cleveland	1871
Coburg	1904
Cologne	1877,1898
Copenhagen	1907,1916,1924,1934
Dallas	1957
Debrecen	1925
Dortmund	1928,1973,1975-1989,1991-2007
DosHermanas	1991-1997,1999,2001,2003,2005
Dresden	1892,1926
Duisburg	1929
Dundee	1867
Dusseldorf	1862,1908
Enghien	2003
Foros	2007
Frankfurt	1878,1887,1923,1930
Geneva	1977
Giessen	1928
Gijon	1944,1945
Gothenburg	1909,1920
Groningen	1946
Hague	1928
Hamburg	1885,1910,1921
Hannover	1902
Hastings	1895,1919,1922,1923,1925-1927,1929-1938,1945,1946,1949,1950,1953,<br>1954,1957,1959-1962,1964,1966,1969-2004
Havana	1913,1962,1963,1965
Heidelberg	1949
Hilversum	1973
Hollywood	1945
Homburg	1927
Hoogeveen	2003
Johannesburg	1979,1981
Karlovy	1948
Karlsbad	1911,1923,1929

Continued on next page

Table S1 – continued from previous page

Open	Years
Kecskemet	1927
Kemeri	1937,1939
Kiel	1893
Kiev	1903
Krakow	1940
Kuibyshev	1942
LakeHopatcong	1926
LasPalmas	1973-1978,1980-1982,1991,1993,1994,1996
Leiden	1970
Leipzig	1876,1877,1879,1894
Leningrad	1934,1937,1939
Leon	1996
Liege	1930
Linares	1981,1983,1985,1988-1995,1997-2007
Ljubljana	1938
Ljubojevic	1975,1977
Lodz	1907,1935,1938
London	1862,1866,1872,1876,1877,1883,1892,1900,1922,1927,1932,1946,1980, 1982,1984,1986
LosAngeles	1963
Lugano	1970
Lviv	2000
Madrid	1943,1996,1997,1998
Maehrisch	1923
Magdeburg	1927
Manchester	1857,1890
Manila	1974, 1975
Mannheim	1914
MardelPlata	1928,1934,1936,1942-1957,1959-1962,1965-1972,1976,1979,1981,1982
Margate	1935,1939
Marienbad	1925
Meran	1924
Merano	1926
Merida	2000,2001
Milan	1975
MonteCarlo	1901-1904,1967
Montecatini	2000
Montevideo	1941
Montreal	1979
Moscow	1899,1901,1920,1925,1935,1947,1956,1966,1967,1971,1975,1981,1985, 1992,2005-2007
Munich	1900,1941,1942,1993
Netanya	1968,1973
NewYork	1857,1880,1889,1893,1894,1913,1915,1916,1918,1924,1927,1931,1940, 1951
Nice	1930
Niksic	1978,1983

Continued on next page

Table S1 – continued from previous page

**Open**	**Years**
Noordwijk	1938
Nottingham	1936
Novgorod	1994-1997
NoviSad	1984
Nuremberg	1883,1896,1906
Oslo	1984
Ostende	1905-1907,1937
Palma	1967,1968,1970,1971
Paris	1867,1878,1900,1924,1925,1933
Parnu	1937,1947,1996
Pasadena	1932
Philadelphia	1876
Podebrady	1936
Poikovsky	2004-2007
Polanica	1998,2000
Portoroz	1985
Prague	1908,1943
Ramsgate	1929
ReggioEmilia	1985-1989,1991,1992
Reykjavik	1987,1988,1991
Riga	1995
Rogaska	1929
Rosario	1939
Rotterdam	1989
Rovinj	1970
Salzburg	1943
SanAntonio	1972
SanRemo	1930
SanSebastian	1911,1912
SantaMonica	1966
Sarajevo	1984,1999,2000
Scarborough	1930
Semmering	1926
Skelleftea	1989
Skopje	1967
Sliac	1932
Sochi	1973,1982
Sofia	2005,2007
SovietChamp	1920,1923-1925,1927,1929,1931,1933,1934,1937,1939,1940,1944,1945,1947-1953,1955-1981,1983-1991
StLouis	1904
StPetersburg	1878,1895,1905,1909,1913
Stepanakert	2005
Stockholm	1930
Stuttgart	1939
Sverdlovsk	1943
Swinemunde	1930,1931

Continued on next page

Table S1 – continued from previous page

**Open**	**Years**
Szcawno	1950
Teeside	1975
Teplitz	1922
TerApel	1997
Tilburg	1977-1989,1991-1994,1996-1998
Titograd	1984
Trencianske	1941,1949
Triberg	1915,1921
Turin	1982
Ujpest	1934
Vienna	1873,1882,1898,1899,1903,1907,1908,1922,1923,1937,1996
Vilnius	1909,1912
Vinkovci	1968
Vrbas	1980
Waddinxveen	1979
Warsaw	1947
WijkaanZee	1968-2007
Winnipeg	1967
Zagreb	1965
Zandvoort	1936
